# Epidemiological profile of the Ebola virus disease outbreak in Nigeria, July-September 2014

**DOI:** 10.11604/pamj.2015.21.331.5834

**Published:** 2015-08-31

**Authors:** Emmanuel Onunche Musa, Elizabeth Adedire, Olawunmi Adeoye, Peter Adewuyi, Ndadilnasiya Waziri, Patrick Nguku, Miriam Nanjuya, Bisola Adebayo, Akinola Fatiregun, Bassey Enya, Chima Ohuabunwo, Kabiru Sabitu, Faisal Shuaib, Alex Okoh, Olukayode Oguntimehin, Nnanna Onyekwere, Abdulsalami Nasidi, Adebola Olayinka

**Affiliations:** 1 World Health Organization, Country Office, Abuja, Nigeria; 2 Nigeria Field Epidemiology and Laboratory Training programme, Abuja; 3 World Health Organization, Department of Communicable Diseases, Kampala District, Uganda; 4 Department of Community Medicine, Lagos State University Teaching Hospital, Ikeja, Lagos, Nigeria; 5 World Health Organisation, Field Office, State Ministry of Health, Akure, Ondo State, Nigeria; 6 Department of Community Medicine, Ahmadu Bello University, Zaria, Nigeria; 7 Federal Ministry of Health, Abuja, Nigeria; 8 Lagos State Primary Health Care Board, Lagos, Nigeria; 9 Rivers State Ministry of Health, Port Harcourt, Nigeria; 10 Nigeria Center for Disease Control, Abuja, Nigeria

**Keywords:** Epidemiological profile, Ebola virus disease outbreak, Nigeria

## Abstract

**Introduction:**

In July 2014, Nigeria experienced an outbreak of Ebola virus disease following the introduction of the disease by an ill Liberian Traveler. The Government of Nigeria with the support of Technical and Development Partners responded quickly and effectively to contain the outbreak. The epidemiological profile of the outbreak that majorly affected two States in the country in terms of person, place and time characteristics of the cases identified is hereby described.

**Methods:**

Using field investigation technique, all confirmed and probable cases were identified, line-listed and analysed using Microsoft Excel 2007 by persons, time and place.

**Results:**

A total of 20 confirmed and probable cases; 16 in Lagos (including the index case from Liberia) and 4 in Port Harcourt were identified. The mean age was 39.5 ± 12.4 years with over 40% within the age group 30-39 years. The most frequent exposure type was direct physical contact in 70% of all cases and 73% among health care workers. The total case-fatality was 40%; higher among healthcare workers (46%) compared with non-healthcare workers (22%). The epidemic curve initially shows a typical common source outbreak, followed by a propagated pattern.

**Conclusion:**

Investigation revealed the size and spread of the outbreak and provided information on the characteristics of persons, time and place. Enhanced surveillance measures, including contact tracing and follow- up proved very useful in early case detection and containment of the outbreak.

## Introduction

Ebola virus disease (EVD) is one of the world's most virulent diseases with high infectivity and case-fatality rate of up to 90% [1–5]. Most previous EVD outbreaks occurred in rural communities with case counts and fatalities ranging from 17 to 318 and 50 to 90% respectively [3–8]. The current outbreak of EVD in West Africa, the largest ever reported globally, began in Guinea in December 2013 [3, 5]. The World Health Organization (WHO) was notified officially in March 2014 and in August declared the epidemic a Public Health Emergency of International Concern (PHEIC). As of November 11 2014, a total of 14,413 confirmed and probable cases as well as 5,777 deaths from eight countries: Guinea, Liberia, Mali, Nigeria, Senegal, Spain, Sierra Leone and the USA are reported [5]. Ebola virus is transmitted by direct contact with blood, body fluids, tissues and corpses of infected animals or people [1, 3]. Outbreaks of EVD are contained using available interventions, like early detection and isolation, contact tracing and monitoring, public enlightenment and adherence to rigorous procedures of infection-control [1, 5–8]. The Federal Ministry of Health (FMoH) was notified of a suspected case of EVD on July 22 2014; based on the symptoms, signs and deteriorating condition with travel history of a patient in a private hospital on 20 July 2014 in Nigeria. The case was confirmed as EVD on 23 July. Immediately an Ebola Emergency Operation Centre (EEOC) was set up by the Federal Ministry of Health and the Lagos State Government in collaboration with technical partners to coordinate all activities in response to the outbreak. We describe the investigation of the index case, the subsequent identification of additional cases and the epidemiologic description by person, place and time characteristics of confirmed cases identified during the outbreak.

## Methods

**Outbreak locations**: Nigeria a country in West Africa, with over 170 million people shares land borders with the Republic of Benin in the west, Chad and Cameroon in the east, Niger in the north and the Gulf of Guinea in the south. Nigeria is located in the tropics. There are 37 States and 6 geopolitical zones namely; Northwest, Northeast, Northcentral, Southwest, Southeast and Southsouth [9]. The outbreak occurred in two States, one in the southwest, Lagos State and the other in the Southsouth, Rivers State. Lagos State is an administrative division of Nigeria, the smallest in land area among all States in the country. It has a population of over 17 million people, it's the nation′s largest urban population and is arguably the most economically important state of the country. In the West it shares boundaries with the Republic of Benin. Behind its southern borders lies the Atlantic Ocean. About 22% of its 3,577 km^2^ are lagoons and creeks. Rivers State's capital Port Harcourt is bounded on the South by the Atlantic Ocean and has a total population of over 6.7 million people. The inland part of Rivers state consists of tropical rainforest; towards the coast the typical Niger Delta environment features many mangrove swamps [2, 9].

**The index case investigation**: the index case was a traveler from Liberia to Lagos who arrived ill and was taken to a private health facility, where he was admitted with fever, vomiting and diarrhea. An initial diagnosis of suspected EVD was made. Laboratory analysis of his blood and urine samples confirmed EVD on 23rd July 2014. The Federal Ministry of Health was notified. The patient however died on the 25th July 2014.

**Field investigation**: the investigation team comprising Nigeria Field Epidemiology and Laboratory Training Programme (NFELTP) residents, volunteers, UNICEF, WHO, Local, State and National Government officials; met after the confirmation of the index case. The team working at the EEOC planned the administrative, consultative and logistic measures. Visits were made to health facilities and homes to identify contacts of suspected, probable and confirmed cases or deaths where the outbreak occurred or was rumored to have occurred As part of active surveillance and containment measures undertaken, contact tracing and follow up measures were adapted from the WHO guidelines [3, 6, 10–13]. A contact was defined as any person without any disease symptoms and/or signs but who had physical contact with or touched the body fluids, or contaminated materials used by a confirmed case, or slept, ate, or spent time in the same household or room of a case (alive or dead), within the preceding three weeks. Physical contact includes sharing the same room/bed, caring for a patient, touching, shaking hands or closely participating in a burial. All persons meeting the definition of a contact were listed. These contacts were subsequently followed up every day for 21days and observed for symptoms, including development of fever (37.50C axillary temperature) becoming a case, or otherwise discarded. To identify cases or deaths from EVD, we defined a suspected case as any person with acute onset of fever, malaise, myalgia, headache, followed by vomiting, diarrhea, and may or may not be accompanied by any of: maculopapular rash, pharyngitis, bloody diarrhea, bleeding from the gums, bleeding under the skin (purpura), bleeding into the eyes (conjunctiva hemorrhage), blood in the urine (haematuria), with no known predisposing hemorrhagic condition. A probable case was defined as a deceased suspected case (where it has not been possible to collect specimen for laboratory confirmation) but has an epidemiological link with a confirmed case Whereas, a confirmed case was defined as a case with clinical illness or a probable case with laboratory confirmation of Ebola virus infection by RT-PCR We established the existence of an outbreak with a single confirmed case.

**Enhanced surveillance**: enhanced surveillance activities included active case search in schools, places of worship, health facilities and community as well as screening of passengers at the points of entry for EVD;. In the health care facilities, we monitored HCW deaths, illness, or sick leave or unexplained absenteeism.

**Contact tracing**: contact listing form and contact follow-up forms were used for listing of contacts and follow up. The contact listing form obtained information on name, age, sex, address, contact, and phone numbers. The contact follow-up form obtained information on name, age, sex, address, date of last contact, type of contact, household information, phone numbers and clinical data of contacts. GPS location of contacts was also mapped using GPS enabled Smartphone devices.

**Points of entry**: screening of passengers at points of entry took place at the air/sea ports and land border crossings. Using Infra red thermometer body temperature of all passengers were taken and recorded on the Health Screening Form (HSF). The number of flights/vessels was collected and the details of any passengers referred for secondary screening. At the airports, this was termed primary data and captured in registers at P1 stations at arrival and P2 stations at departure. The number of passengers screened daily was calculated by counting the number of screening forms collected at each of these stations. This was collected and collated by a data manager every morning. At the seaport data on number of vessels and travelers under surveillance was collated using the HSFs and simple temperature monitoring charts. The data was transmitted to the team lead using data enabled mobile phones configured with a data reporting template.

**Data analysis**: a line-list of cases was created consisting of data on the date of onset of illness, age, sex, occupation, exposure type, presenting symptoms and outcomes obtained and updated. Using the line-list, an epidemic curve was plotted with the number of cases and deaths against the date of onset of disease symptoms. The median incubation period was estimated from the curve as the time interval between the arrival of the index case and the date of the peak of occurrence of cases on the epidemic curve. The serial interval was defined as the time of disease onset in the index case and onset in a person infected by the index case. The characteristics of cases stratified by occupation and the presenting symptoms were presented in tables. Microsoft Excel 2007 was used in analyzing the data.

**Sites for case management**: in Lagos, an area within the existing Infectious Disease Hospital was converted to the Ebola Treatment Centre (ETC). This 40-bed capacity isolation facility has separate sections for suspected and confirmed cases. The Lagos clinical staff include 15 doctors, 28 nurses and 16 ancillary staff trained on Ebola case management in the isolation facility and they provided 24-hour care alongside the WHO and MSF staff. In Port Harcourt, the State Ministry of Health (SMOH) a former primary healthcare center was converted into a 26-bed isolation facility with separate sections for suspected and confirmed cases. The clinical team included MSF and SMOH doctors (12), nurses (24) and support staff (24) who were all trained on Ebola case management and appropriate infection prevention and control in the isolation facility. These facilities operated with Laboratory support from the Virology Laboratory of the Lagos University Teaching Hospital (LUTH), and an European Union-donated mobile PCR laboratory supported by WHO in Rivers state.

## Results

**Description of the index case**: the index case was a 40-year old male Liberian-American who came to Nigeria on 20th July 2014 to attend the 8th ECOWAS Retreat of Heads of Offices meeting in Calabar, Cross River State. He was reported to have been ill while on board a flight from Monrovia to Lagos and remained very ill on arrival at the airport. He was admitted at a private facility with fever, vomiting and diarrhea. While fully conscious, he reported no history of contact with anyone suspected or confirmed to be suffering from EVD in Liberia. Later he was discovered to have visited and cared for a sibling in Liberia who died of EVD with his symptoms starting 17th of July 2014.

**Figure 1:**
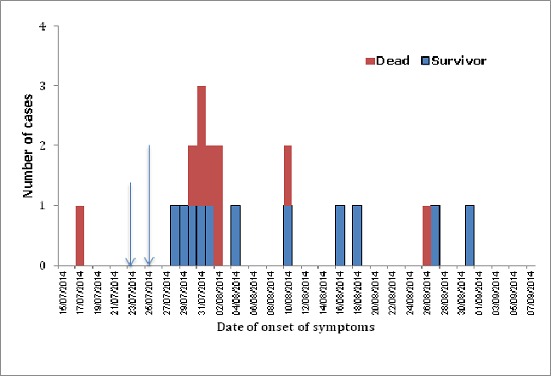
Epidemic curve of Ebola virus disease in Nigeria, July-September 2014

**Outcome of contact tracing and identification of cases**: a total of 899 contacts were identified and followed up, 362(40.3%) from Lagos, 530(58.9%) from Port Harcourt and 7(0.8%) from Enugu; from these, 19 laboratory confirmed and one probable case were recorded, 16 (including one probable case) in Lagos and 4 in Port Harcourt (Figure 1). There were 24 suspected cases; 16 in Lagos and 8 in Port Harcourt that were not confirmed as EVD by the laboratory. As of September 30, 2014, the total numbers of passengers screened at the points of entry were 599,204 in Lagos and 124,903 in Port Harcourt, with no EVD case detected.

**Figure 2:**
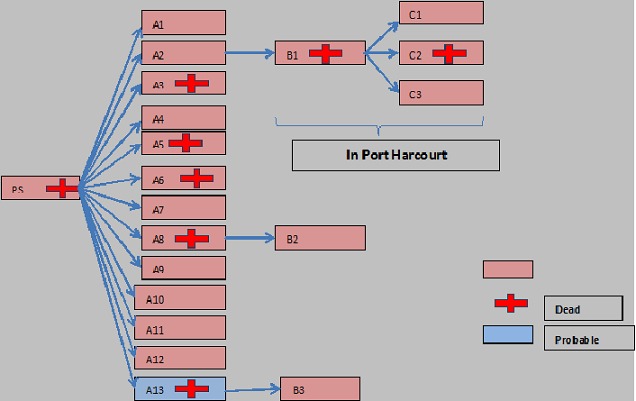
Transmission chain of cases of Ebola disease outbreak in Nigeria, July-September, 2014; A): primary generation cases; B): secondary generation cases; C):tertiary generation cases

**Description of cases**: the characteristics of the cases, in terms of age, sex, exposure types and outcomes are shown in Table 1. The mean age was 39.5± 12.4 years with over 40% within the age group 30-39 years. Majority (55%) of cases were females and health workers. The most frequent exposure type was direct physical contact in 70% of cases and 73% among health workers. Fever (60%), fatigue, vomiting and diarrhoea (40%) were the most reported symptoms at presentation. Only 5% (1) presented with any form of bleeding diathesis (Table 2). The total case-fatality was 40%; higher among healthcare workers (46%) compared to non healthcare workers (22%). The epidemic curve (Figure 1) initially shows a typical common source outbreak, followed by a propagated pattern; though atypical. The total length of epidemics was 43 days. The median incubation period was 11days and serial interval for the first wave of infection was 11days. Arising from the primary chain of transmission were secondary and tertiary generations of cases (Figure 2).

**Table 1:** Characteristics of Ebola virus disease cases, July-September, 2014

Characteristics	Health worker (n = 11,55%)	%	Non Health Worker (n = 9, 45%)	%	Total (n = 20)	%
**Age (Years)**						
20-29	3	27	1	11	4	20
30-39	4	36	4	44	8	40
40-49	1	9	2	22	3	15
50-59	2	18	1	11	3	15
≥60	1	9	1	11	2	10
***Mean Age = 39.5 years Standard Deviation = 12.4 years***						
**Sex**						
Male	5	46	4	44	9	45
Female	6	54	5	56	11	55
**Type of Exposure**						
*Touching body fluids	1	9	2	22	3	15
Direct physical contacts	8	73	6	67	14	70
Manipulation of infected objects	1	9	0	0	1	5
Was in the same room case	1	9	1	11	2	10
**Place of Residence**						
Lagos	9	82	7	78	16	80
Port Harcourt	2	18	2	22	4	20
**Outcome**						
Discharged	6	50	6	50	12	60
Dead	5^a^	46	2^b^	22	7^c^	35

a Case Fatality Rate (Health Worker) = 45.5%,

b Case Fatality Rate (Non Health Worker) = 22.2%,

c Case Fatality Rate (Total) = 35%,

* Generally understood as blood, urine, saliva, vomitus, stool

**Table 2:** Clinical features of Ebola Virus Disease cases in Nigeria, July-September, 2014

Clinical features at presentation	+Frequencies	Percent (%)
Fever	12	60
Fatigue	8	40
Vomiting	8	40
Diarrhea	8	40
Loss of appetite	7	35
Headache	3	15
Joint Pain	3	15
Conjunctivitis	2	10
Muscle Pain	2	10
Confusion	1	5
Eye pain	1	5
Any bleeding tendencies	1	5

+ Multiple response

## Discussion

With a total of twenty cases the size of the current outbreak in the country is small compared to the concurrent outbreak in Guinea, Sierra Leone and Liberia [2, 3, 5]. However, in terms of spread, cases were identified in two major urban cities that are geographically far from each other. This contrasts with the current outbreaks reported in Guinea, Liberia and Sierra Leone where contiguous districts and communities were affected [5]. This mode of spread by air travel has not been observed in any of the previous EVD outbreaks, and it may have contributed to the current declaration of PHEIC by the WHO [5, 8]. In addition, the length of the outbreak in Nigeria was 43 days; shorter than most outbreaks previously and currently reported [5, 10, 11–14]. This suggests efficient containment. The case fatality in Nigeria was far less than the 70.8% seen in Liberia, Guinea and Sierra Leone [5]. This pattern of lower case fatality was also observed among health workers, who are the high-risk group in all the other EVD outbreaks reported including the concurrent outbreaks in other West African countries suggesting an effective response [1, 5]. As observed in previously reported outbreaks from other African countries, including the concurrent outbreak in West Africa sub region, females were the most affected [3–5, 7, 12–15]. This may be explained by the role that the female gender plays in care-giving and nursing in our society, thereby exposing them to infection. [1–5]. Majority of the cases reported direct physical contact with an infected person. This is more obvious among the health workers. Other factors may include the lack of consideration given to potential body fluids which were not overtly visible. A clearer understanding of the role of direct physical contact in the absence of visible bodily fluid is needed through further research as this has implications for the containment of the disease.

The concurrent outbreak in the West Africa sub region similar to our study also indicated that the most productive middle-aged groups are affected [5]. Fever, fatigue, vomiting and diarrhoea were the most commonly reported symptoms at presentation. Only very few presented with any form of bleeding diathesis. These are consistent with clinical symptoms being reported from the concurrent epidemics in other countries within the sub region [5, 11–14]. The epidemic curve initially shows a typical common source pattern, followed by a propagated; though atypical. This is in contrast with the epidemic curve described in the outbreak in Gabon with a typical propagated pattern [10]. The timely containment measures and the multiple strategies used might have contributed to the atypical pattern observed. The median incubation period we obtained is consistent with the known incubation. Apart from the primary wave of infection there were two other waves; secondary and tertiary in the current outbreak. The serial interval for the first wave was 11days. We were not able to explore the data further to generate the reproductive rate due to the small number of cases. We recognize the challenges that the field investigation may have imposed on our findings. Firstly, the outbreak locations were in two geographical areas that are wide apart. This could have imposed coordination difficulties and standardization of procedures and observations, but same protocols and SOPs were used in the 2states and all data were sent to the same server. Secondly, the teams of public health workers used in the response had no previous experience in response to EVD outbreaks although they were highly skilled health professionals. However, misclassification bias was minimized by using a standard case definition during case identification. Ascertainment of the accuracy of the data was performed at every stage of the data collection process.

## Conclusion

Enhanced surveillance measures, including contact tracing and follow-up, effective case management and social mobilization efforts with effective coordination of government and partner agencies proved very useful in containment of this outbreak
